# Mirtazapine-induced bilateral secondary angle closure in a female

**DOI:** 10.3205/oc000156

**Published:** 2020-06-29

**Authors:** Srishti Raj, Manpreet Kaur, Kandragunta Srinivasarao, Faisal Thattaruthody, Sushmita Kaushik, Surinder Singh Pandav

**Affiliations:** 1 Advanced Eye Centre, Post-Graduate Institute of Medical Education and Research, Chandigarh, India

**Keywords:** mirtazapine, secondary angle closure, ciliary body edema, side effects

## Abstract

**Purpose:** To report a case of bilateral secondary angle closure in a female using mirtazapine for 6 months.

**Patient and method:** A 55-year-old female was diagnosed with secondary angle closure in both eyes with raised intraocular pressure, and ultrasound biomicroscopic findings suggestive of ciliary body effusion. It was associated with adjoining cyst presumably because of the use of mirtazapine for depression and sleep disturbances.

**Results:** After the planned discontinuation of mirtazapine, the ocular angle opened, the ciliary body edema decreased, and the cyst regressed in size. The intraocular pressure was controlled with topical timolol (0.5%).

**Conclusion:** Patients with risk factors for angle closure should be prescribed antipsychotic drugs with caution. The peripheral laser iridotomy is not indicated in secondary angle closure due to ciliary body effusion.

## Introduction

A number of systemic and local medications have a mydriatic effect on the pupil. The common drugs causing angle closure in some anatomically predisposed eyes are anticholinergics, antipsychotics, anticonvulsants, adrenergics, mydriatics, antihistamines and anti-sulfamate derivatives [1].

Mirtazapine, an antipsychotic drug, is a newer drug used for the management of moderate to severe depression worldwide [2]. There is scarce literature suggesting its association with angle closure glaucoma [3]. We here describe ocular findings of the secondary angle-closure in a middle-aged female using mirtazapine for depressive illness and sleep disturbances.

## Case description

A 55-year-old female presented with a 6-month history of diminution of vision in both eyes with intermittent headache. She had been diagnosed with a depressive illness for which mirtazapine tablets 15 mg/day had been prescribed 6 months earlier. On examination, her best-corrected visual acuity was 6/18 in both eyes. The intraocular pressure measured with Goldmann applanation tonometer was 22 mm Hg and 28 mm Hg in the right and the left eye, respectively, without any anti-glaucoma medications. The slit-lamp examination revealed a shallow anterior chamber in both eyes with Van Herick grade 1. The patient’s posterior segment examination revealed a cup-disk ratio of 0.4 and 0.6 in the right and the left eye, respectively, with healthy neuro-retinal rim.

**Figure 1:**
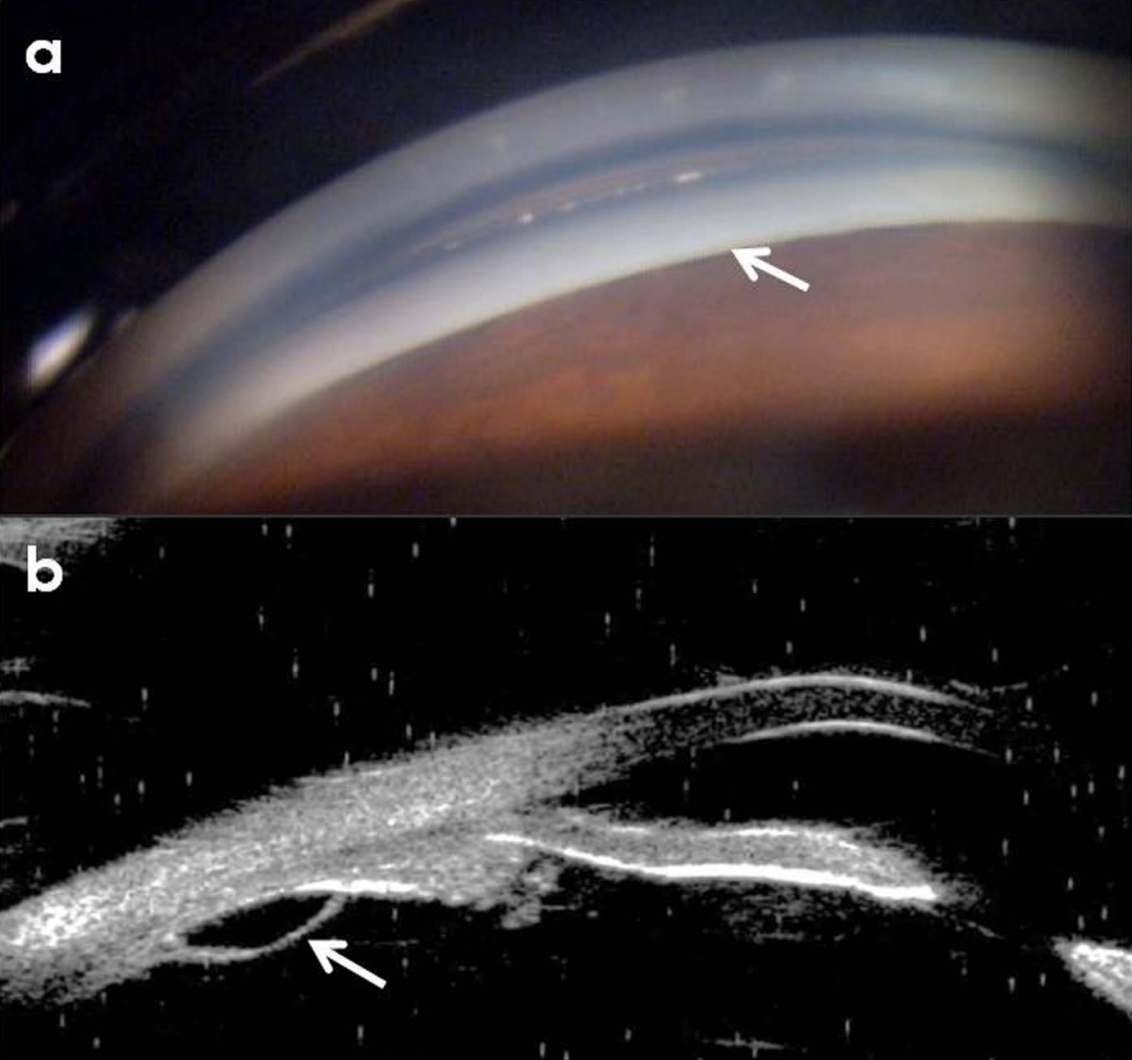
a) Gonioscopy showing closed angles (Shaffer’s grade 0) b) Ultrasound biomicroscopy picture showing ciliary body edema with adjoining cyst

The Zeiss 4-mirror gonioscopy revealed closed angles in both eyes (Shaffer’s grade 0) (Figure 1a (Fig. 1)). The patient’s central corneal thickness was 536 µ and 542 µ in the right and the left eye, respectively. Her ultrasound biomicroscopic examination (UBM) revealed an edematous ciliary body in both eyes measuring 2.1 mm from the inner point of ciliary processes to the inner wall of the sclera with an adjoining cyst (Figure 1b (Fig. 1)). Topical timolol maleate 0.5% BD was started in both eyes, and the patient was diagnosed as a case of mirtazapine-induced secondary angle closure.

**Figure 2:**
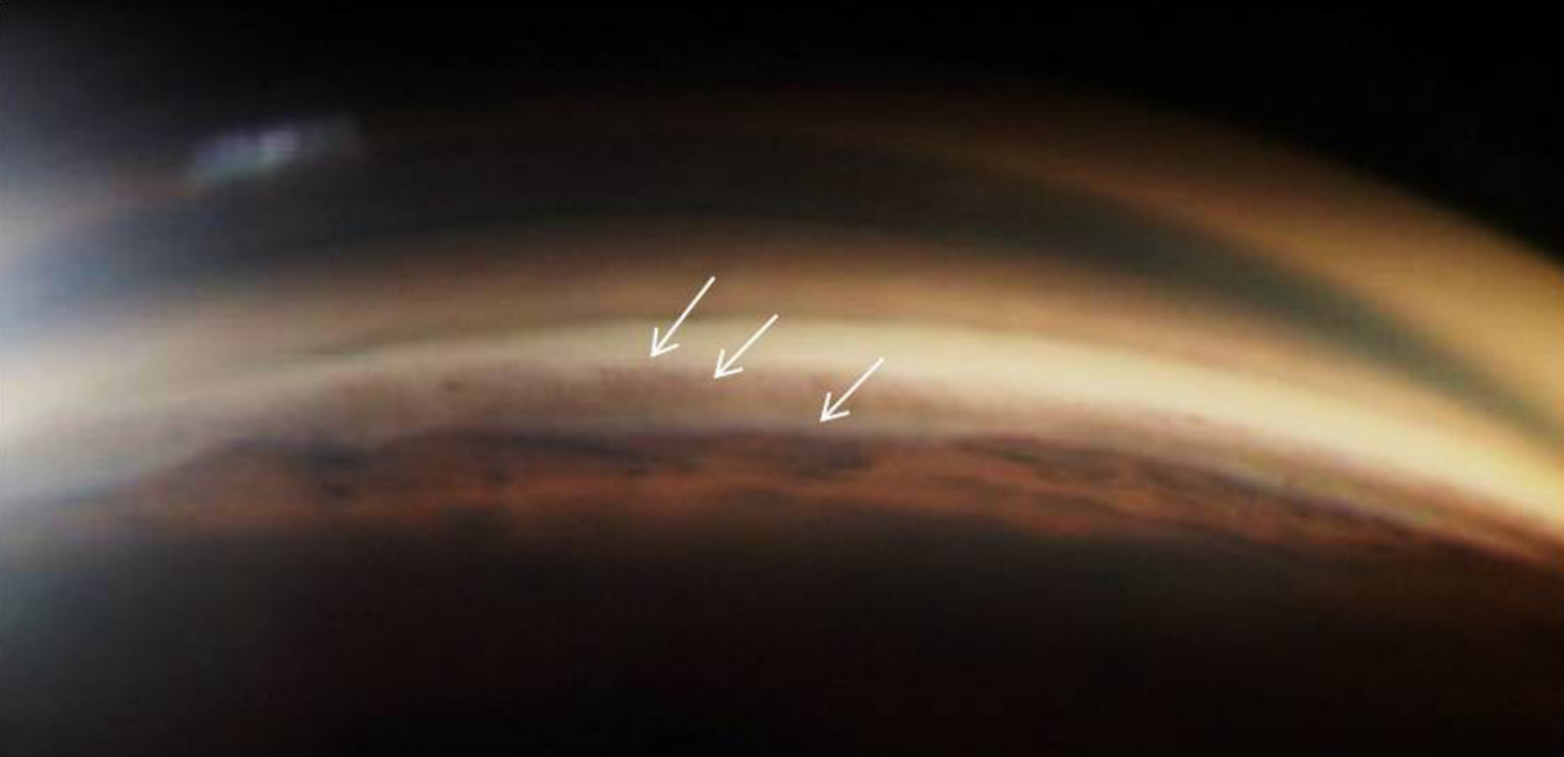
Gonioscopy after stopping mirtazapine shows open angles (Shaffer’s grade 3)

After psychiatric consultation, mirtazapine was changed to sertraline 50 mg/day. After 3 months, the intraocular pressure dropped to 16 mm Hg in BE (on timolol) and angles were open on gonioscopy (Shaffer’s grade 3) (Figure 2 (Fig. 2)). The ciliary body edema decreased from 2.1 mm to 1.78 mm with regression of the adjoining cyst. At present, the patient is on follow-up for monitoring the ciliary body edema and gonioscopy.

## Discussion

A number of case reports in the literature have highlighted various antipsychotic drugs which are known to cause angle-closure glaucoma either with pupillary block or non-pupillary block mechanisms (cilio-choroidal effusion, ciliary body edema) [4], [5].

Mirtazapine acts by enhancing noradrenergic and 5-HT1A-mediated serotonergic neurotransmission by acting as an antagonist at the central a2-adrenergic autoreceptors and heteroreceptors, as well as by post-synaptic blockade of 5-HT2 and 5-HT3 receptors [2].

Various risk factors like shorter axial length increase lens thickness, and shallow anterior chamber increases the chance of angle closure. Peripheral laser iridotomy is indicated only if the mechanism is pupillary block.

To the best of our knowledge, there is a single case report of mirtazapine-induced angle closure after single dose of the drug (15 mg/day) [3]. In that report, the authors highlighted the “mydriatric effect” of the drug as a possible mechanism of acute angle closure. In contrast, we present a well-documented angle-closure effect of oral mirtazapine with ciliary body effusion as a possible mechanism.

In our case, the patient was asymptomatic, and only on detailed examination could we find the association between her clinical features and mirtazapine. The ciliary body edema with adjoining cyst suggests some mechanism other than mydriasis might be responsible for angle-closure glaucoma, as discontinuation of the drug resulted in an improvement in the form of open angles on gonioscopy. The pathophysiology remains unclear; an idiosyncratic reaction of some drugs that resulted in ciliary body edema has been reported in the literature [5].

## Conclusion

Angle-closure glaucoma induced by these antipsychotic drugs can result in serious or blinding complication if not recognized early. Clinicians should be cautious prescribing these medications to patients, taking into account the risk factors like female sex, hyperopia, and family history of glaucoma.

## Notes

### Competing interests

The authors declare that they have no competing interests.

### Informed consent

The patient has given written consent to publish the case report.
